# Serum Carotenoids Reduce Progression of Early Atherosclerosis in the Carotid Artery Wall among Eastern Finnish Men

**DOI:** 10.1371/journal.pone.0064107

**Published:** 2013-05-21

**Authors:** Jouni Karppi, Sudhir Kurl, Kimmo Ronkainen, Jussi Kauhanen, Jari A. Laukkanen

**Affiliations:** 1 University of Eastern Finland, Department of Medicine, Institute of Public Health and Clinical Nutrition, Kuopio, Finland; 2 Department of Internal Medicine, Lapland Central Hospital, Rovaniemi, Finland; John Hopkins Bloomberg School of Public Health, United States of America

## Abstract

**Background:**

Several previous epidemiologic studies have shown that high blood levels of carotenoids may be protective against early atherosclerosis, but results have been inconsistent. We assessed the association between atherosclerotic progression, measured by intima-media thickness of the common carotid artery wall, and serum levels of carotenoids.

**Methods:**

We studied the effect of carotenoids on progression of early atherosclerosis in a population-based study. The association between concentrations of serum carotenoids, and intima-media thickness of the common carotid artery wall was explored in 840 middle-aged men (aged 46–65 years) from Eastern Finland. Ultrasonography of the common carotid arteries were performed at baseline and 7-year follow-up. Serum levels of carotenoids were analyzed at baseline. Changes in mean and maximum intima media thickness of carotid artery wall were related to baseline serum carotenoid levels in covariance analyses adjusted for covariates.

**Results:**

In a covariance analysis with adjustment for age, ultrasound sonographer, maximum intima media thickness, examination year, body mass index, systolic blood pressure, smoking, physical activity, serum LDL cholesterol, family history of coronary heart disease, antihypertensive medication and serum high sensitivity C-reactive protein, 7-year change in maximum intima media thickness was inversely associated with lycopene (p = 0.005), α-carotene (p = 0.002) and β-carotene (p = 0.019), respectively.

**Conclusions:**

The present study shows that high serum concentrations of carotenoids may be protective against early atherosclerosis.

## Introduction

It is well known that early development atherosclerosis is closely associated with lipoprotein metabolism via oxidative modification of low-density lipoprotein (LDL). The presence of oxidative modified LDL in the subendothelium of arteries affect monocyte differentiation to macrophages leading to the formation of foam cells and increased thickness of the artery wall [1], [2]. Macrophages bind and take up oxidative modified LDL particles via scavenger receptors, but not un-oxidized, native LDL particles [3].

Carotenoids, abundant in many fruits and vegetables, are plant derived fat-soluble pigments that possess antioxidant activity. They may protect against chronic atherosclerotic diseases by decreasing the oxidative damage of cell lipids, lipoproteins, proteins and DNA [4], [5]. Carotenoids inhibit oxidative modification of LDL and may play a protective role in the development of cardiovascular diseases [6] by preventing the formation of early atherosclerotic lesions [7].

We previously reported that high plasma concentrations of β-cryptoxanthin, lycopene and α-carotene are associated with decreased carotid atherosclerosis in elderly men [8]. In other previous studies, lutein, β-cryptoxanthin and zeaxanthin were inversely associated with progression of atherosclerosis when measuring carotid intima-media thickness (IMT) [9], [10]. Another study suggested that serum lutein may play a protective role in the prevention of early atherosclerosis [11]. Some studies have shown that high serum levels of lycopene may play a protective role against cardiovascular diseases, in particular the carotid atherosclerosis [12], [13]. However, there is still a very limited amount of data showing the role of serum carotenoids in the prevention of early atherosclerosis. Therefore, the aim of the present study was to examine the association between serum levels of carotenoids and atherosclerotic progression, measured by CCA-IMT.

## Methods

### Study population

The Kuopio Ischaemic Heart Disease Risk Factor Study (KIHD) is a population-based, cohort that was designed to identify a wide range of biological, behavioral, socioeconomic, and psychosocial risk factors for cardiovascular disease, diabetes, and other outcomes in a sample of middle-aged men in Kuopio, Finland and its rural communities [14]. The study has been approved by the Research Ethics Committee of the Hospital District of Northern Savo, Kuopio, Finland. All study subjects gave their written informed consent. Baseline study was carried between March 1991 and December 1993. Of a total of 1229 men eligible for the baseline study, 35 had died, 12 were suffering severe illness, 5 had migrated away from the region, 2 had no address and 137 refused to participate. Thus, 1038 men were participated in the baseline study. Reexaminations were conducted between March 1998 and February 2001 (7 years follow-up). Of a total of 1173 men eligible for the re-examination study, 14 had died, 97 were suffering severe illness, 10 had migrated away from the region, 3 had no address, 168 had refused, 51 had no contact and 34 had other reason not to participate. Of a total of 920 participants, high-resolution ultrasound examinations of CCA-IMT and data on serum carotenoid concentrations were available for 840 men.

### Ultrasonographic assessment of the intima-media thickness of the common carotid artery wall

The extent of carotid atherosclerosis was assessed by high-resolution B-mode ultrasonography of the right and left common carotid arteries (CCAs) in a 1.0 to 1.5 cm section at the distal end of the CCA proximal to the carotid bulb. Images were focused on the posterior wall of the right and left CCAs and recorded on videotape. Ultrasound examinations were conducted by 1 of 4 trained sonographers and were performed with the subject lying supine after a 15 min rest period. Both assessments were obtained with a Biosound Phase 2 scanner (BiosoundEsaote) that was equipped with a 10 MHz annular array probe [15]. Details of the scanning procedures, reliability, and precision of measurement have been reported elsewhere [15], [16]. Computerized analysis of videotaped ultrasound images via Prosound software (University of Southern California) was conducted with an edge-detection algorithm [17] permitting automatic detection, tracking, and recording of the intima/lumen and media/adventitia interfaces. IMT, calculated as the mean distance between these interfaces, was estimated at approximately 100 points in both right and left CCAs.

In the present study, we used 3 measures of IMT: mean IMT (IMT_mean_), the mean of all of the IMT estimates from the right and left CCAs; maximum IMT (IMT_max_), the mean of the points of maximum thickness from the right and left CCAs; and plaque height, the average of right and left CCA measurements of plaque height, calculated as the difference between maximum and minimum thicknesses.

### Blood sampling

Blood samples were taken between 8.00 and 10.00 a.m. Blood was collected in Terumo Venoject vacuum serum tubes (10 mL) (Terumo, Tokyo, Japan) from the antecubital vein without tourniquet after an overnight fast. Subjects had rested in a supine position for 30 min before blood sampling. Subjects were instructed to abstain from consuming alcohol for three days and from smoking for 12 hours before blood collection. Serum for carotenoids and other biochemical measurements were divided to other tubes and frozen at −80°C immediately after separation until analysis.

### Analysis of carotenoids

Lycopene, α-carotene and β-carotene serum concentrations were measured from frozen serum that had been stored at −80°C for 4–36 months by using a modification of the high-performance liquid chromatographic method of Thurnham et al. [18]. Briefly, 200 µL of serum was extracted with 5 mL of hexane and 1 mL of ethanol. The hexane layer was separated and evaporated to dryness under nitrogen at room temperature and the residue was dissolved in 200 µL of the mobile phase (acetonitrile-methanol-chloroform 47∶47∶6, v/v/v). Samples were injected in a C_18_ analytical column at room temperature. Peaks were detected at wavelengths of 470 nm for lycopene, at 454 nm for other carotenoids by a diode array detector (Model 168; Beckman Instruments, San Ramon, CA, USA). The limits of detection for carotenoids were 0.03–0.07 µmol/L. Values below the limit of detection of the assay were marked as 0.00 in the statistical analysis. The inter-assay coefficients of variation (CV) varied from 11.0 to 16.2%.

### Other biochemical measurements

Concentrations of serum LDL cholesterol (LDL-c) and triglycerides were analyzed with enzymatic methods (Thermo Fisher Scientific, Vantaa, Finland). Serum HDL cholesterol (HDL-c) was measured after magnesium chloride dextran sulfate precipitation from the supernatant with enzymatic method (Thermo Fisher Scientific). Serum high sensitivity C-reactive protein (hs-CRP) was measured by the chemiluminescence-immunoassay method using Immulite 2000 analyzer (DPC, Los Angeles, USA).

### Other measurements

Resting blood pressure was measured in the morning by two trained nurses with a random-zero mercury sphygmomanometer (Hawksley, Lancing, United Kingdom). Blood pressure at rest was measured by a nurse with a random 0 mercury sphygmomanometer (Hawksley, United Kingdom; from 8:00 to 10:00 a.m). The measuring protocol included, a supine rest of 5 minutes, 3 measurements in the supine position, 1 measurement in the standing position, and 2 measurements in the sitting position at 5-minute intervals. The mean of 6 systolic and diastolic pressures was used in these analyses [19]. Body mass index (BMI) was computed as the ratio of weight (kilograms) to the square of height (meters). Alcohol consumption was assessed with a structured quantity-frequency method on drinking behaviour over the previous 12 months. Information on chronic diseases was checked during a medical examination by the internist. The family history of coronary heart disease (CHD) was defined as positive if father, mother, sister, or brother of the subject had a history of CHD. Data on education, current medications and smoking status were collected with a self-administered questionnaire and checked by the interviewer. Physical activity was assessed by using a 12-month leisure-time history based on self-reported information about frequency per month over the preceding year, average duration per occasion, and intensity level. Metabolic units were assigned for each activity according to intensity. Diabetes mellitus was defined as a fasting blood glucose level ≥6.7 mmol/L or as a clinical diagnose of diabetes with dietary, oral or insulin treatment. A subject was defined as a smoker if he had ever smoked on a regular basis and had smoked cigarettes, cigars, or a pipe within the past 30 days. The lifelong exposure to smoking was estimated as the product of the number of smoking years and the number of tobacco products smoked daily at the time of examination.

### Statistical analyses

Continuous variables were presented as means (standard deviations in parentheses) and categorical variables as percentages. Means of the continuous variables were compared using the ANOVA and χ^2^ tests were used for categorical variables. The relationship between serum carotenoid levels and atherosclerotic risk factors were analyzed with Spearman rank order correlation coefficients. Subjects were classified into tertiles according to their serum concentrations of carotenoids. The association between serum concentrations of carotenoids and progression of CCA-IMT (IMT_mean_ and IMT_max_) was tested for statistical significance by using covariance analysis. Two different sets of covariates were used. Model 1: age, ultrasound sonographer, IMT_mean_ or IMT_max_, and examination years; Model 2: Model 1+BMI, SBP, smoking, physical activity, serum LDL-c, family CHD history, antihypertensive medication; Model 3: model 2+serum hs-CRP. Linear regression models were used to estimate the association with every 0.01 µmol/L increase of carotenoids and reduction of IMT_max_ (µm/7-years). Tests for statistical significance were two-sided and p<0.05 was taken as the criterion of significance (SPSS Statistics software, version 19.0, Chicago, IL, USA).

## Results

Of the 1229 male participants at the baseline, CCA-IMT at 7-year follow-up was available for 840 men (68.3%). Difference at the baseline between smokers and non-smokers is shown in Table 1. Smokers were younger, had higher SBP, lower BMI and consumed more alcohol. The 7-year progression of IMT_max_ was higher among smokers as compared with non-smokers. Smokers had lower levels of serum α-carotene, but their levels of serum LDL-c and hs-CRP were higher than among non-smokers.

**Table 1:** Baseline characteristics of the men: the Kuopio Ischaemic Heart Disease Risk Factor (KIHD) study.

	Total population	Smokers	non-smokers	
Characteristics	(n = 840)^a^	(n = 201)	(n = 638)	p-value^b^
**Demographic characteristics**				
Age (y)	55.6 (6.6)	54.1 (6.6)	56.1 (6.6)	<0.001
SBP (mmHg)	135 (16)	135 (16)	132 (16)	0.005
DBP (mmHg)	89 (10)	87 (11)	89 (9)	0.018
BMI (kg/m^2^)	27.5 (3.5)	26.9 (3.7)	27.6 (3.4)	0.011
Smokers (%)	24.0			
Smoking (pack-year)^c^	8.8 (21.1)			
Alcohol consumption (g/week)	75.6 (106.7)	102.7 (105.8)	67.0 (105.7)	<0.001
Physical activity (kcal/d)	179.1 (205.9)	173.8 (217.3)	180.9 (202.5)	0.672
**Medical history**				
CHD in family (%)	54.0	52.0	55.0	0.395
Diabetics (%)	6.0	5.0	6.0	0.549
Drug for hypertension (%)	26.0	19.0	28.0	0.015
**CCA-IMT (mm)**				
IMT_mean_ at baseline	0.86 (0.20)	0.86 (0.20)	0.86 (0.19)	0.944
IMT_max_ at baseline	1.19 (0.27)	1.20 (0.31)	1.19 (0.26)	0.893
7-y progression (IMT_mean_)	0.096 (0.16)	0.11 (0.16)	0.091 (0.16)	0.076
7-y progression (IMT_max_)	0.078 (0.24)	0.12 (0.27)	0.065 (0.23)	0.008
**Laboratory data**				
Serum lycopene (µmol/L)	0.16 (0.14)	0.16 (0.15)	0.16 (0.14)	0.828
Serum α-carotene (µmol/L)	0.10 (0.08)	0.081 (0.072)	0.10 (0.081)	<0.001
Serum β-carotene (µmol/L)	0.40 (0.31)	0.37 (0.28)	0.41 (0.32)	0.096
Serum LDL-c (mmol/L)	3.91 (0.83)	4.02 (0.92)	3.88 (0.80)	0.035
Serum HDL-c (mmol/L)	1.11 (0.29)	1.12 (0.31)	1.11 (0.28	0.535
Serum triglycerides (mmol/L)	1.59 (1.04)	1.53 (0.92)	1.60 (1.08)	0.417
Serum hs-CRP (mg/L)	2.90 (5.79)	3.91 (7.71)	2.58 (5.00	0.004

Abbreviations: BMI = body mass index; CCA-IMT = intima-media thickness of the common carotid artery wall; CHD = coronary heart disease; DBP = diastolic blood pressure; hs-CRP = high sensitivity C-reactive protein; HDL = high-density lipoprotein; LDL = low-density lipoprotein; SBP = systolic blood pressure.

a Values are given mean (SD) and percentages.

b p for differences between smokers and non-smokers (one-way ANOVA).

c Pack-year denote the lifelong exposure to smoking, estimated as the product of years smoked and the number of tobacco products smoked daily at the time of examination.

The correlations between serum carotenoid levels and atherosclerotic risk factors are shown in Table 2. All carotenoids correlated inversely with BMI, antihypertensive drugs, serum triglycerides and the marker of chronic inflammation (hs-CRP), although correlation coefficients were low. Lycopene and β-carotene correlated positively with physical activity and serum HDL-c levels, whereas α- and β-carotene correlated directly with serum LDL-c levels.

**Table 2:** Spearman's correlation coefficients among serum levels of carotenoids and atherosclerosis risk factors at baseline: the Kuopio Ischaemic Heart Disease Risk Factor (KIHD) study.

Risk factor	Lycopene	α-carotene	β-carotene
BMI (kg/m^2^)	−0.09*	−0.08*	−0.25*
SBP (mmHg)	−0.08*	−0.05	−0.14*
DBP (mmHg)	−0.05	−0.11*	−0.18*
Smoking (pack-year)	−0.05	−0.15*	−0.13*
Alcohol consumption (g/week)	0.19*	−0.10*	−0.18*
CHD in family	0.004	0.000	0.015
Physical activity (kcal/d)	0.14*	0.048	0.08*
Drug for hypertension	−0.14*	−0.09*	−0.10*
Serum LDL-c (mmol/L)	0.02	0.07*	0.09*
Serum HDL-c (mmol/L)	0.08*	−0.05	0.10*
Serum triglycerides (mmol/L)	−0.10*	−0.08*	−0.27*
Serum hs-CRP (mg/L)	−0.11*	−0.12*	−0.25*

* p<0.05.

Abbreviations as in Table 1.

**Figure 1:**
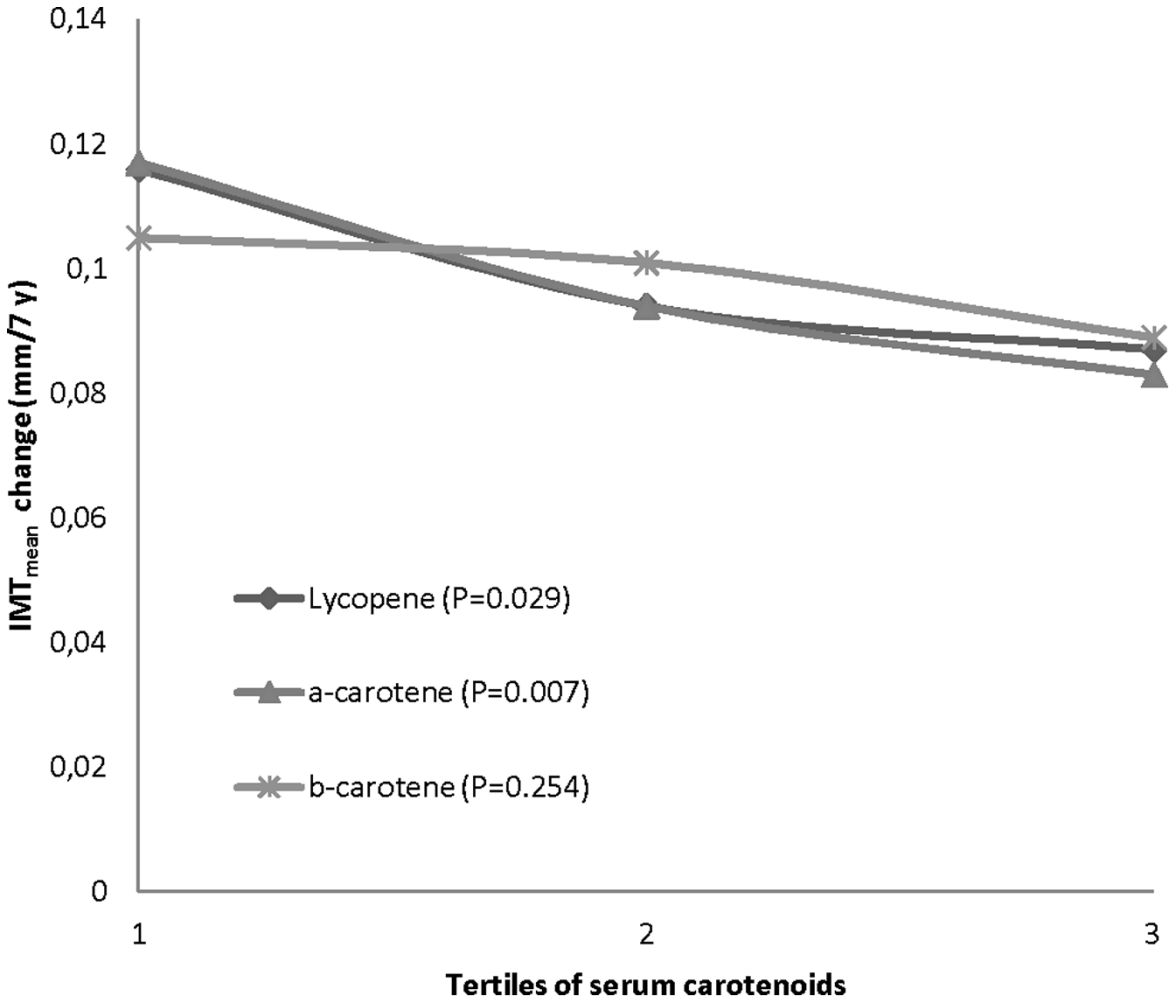
Serum carotenoid tertiles by 7-year IMT_mean_ change (adjusted for age, examination year, ultrasound sonographer, BMI, SBP, IMT_mean_, smoking, serum LDL cholesterol, physical activity, CHD in family, antihypertensive medication and serum hs-CRP. Probability values are for trend. Carotenoid tertiles (µmol/L): lycopene: ≤0.09, 0.10–0.19, ≥0.20; α-carotene: ≤0.07, 0.08–0.11, ≥0.12; β-carotene: ≤0.26, 0.27–0.41, ≥0.42.

**Figure 2:**
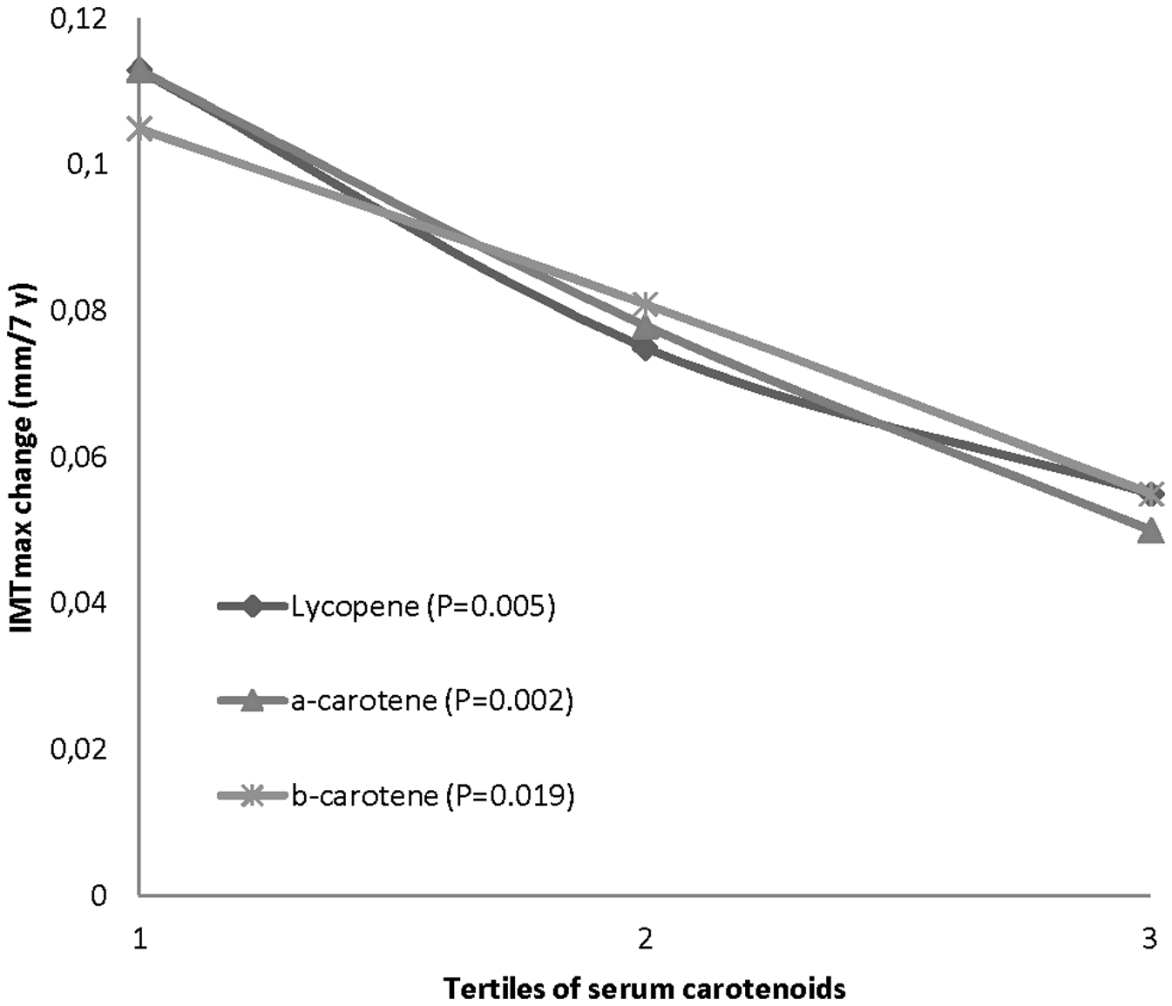
Serum carotenoid tertiles by 7-year IMT_max_ change (adjusted for age, examination year, ultrasound sonographer, BMI, SBP, IMT_max_, smoking, serum LDL cholesterol, physical activity, CHD in family, antihypertensive medication and serum hs-CRP. Probability values are for trend. Carotenoid tertiles (µmol/L): lycopene: ≤0.09, 0.10–0.19, ≥0.20; α-carotene: ≤0.07, 0.08–0.11, ≥0.12; β-carotene: ≤0.26, 0.27–0.41, ≥0.42.

Progression of IMT_mean_ was on average 0.096 mm/7-years and IMT_max_ 0.078 mm/7-years, respectively. Progression of IMT_mean_ and IMT_max_ are presented in Table 3 and Table 4. After adjusting for age, examination year, ultrasound sonographer, IMT_mean_, BMI, SBP, smoking, physical activity, serum LDL-c, family CHD history, antihypertensive medication and serum hs-CRP, higher serum levels of lycopene (p = 0.029) and α-carotene (p = 0.007) were associated with a reduced progression of carotid IMT_mean_. Serum β-carotene had no effect on IMT_mean_ progression. However, after adjusting for corresponding confounders including IMT_max_, higher serum levels of lycopene (p = 0.005), α-carotene (p = 0.002) and β-carotene (p = 0.019) were associated with the reduced progression of carotid IMT_max_. Relationships between the tertiles of carotenoids and atherosclerosis are shown in Figure 1 and Figure 2.

**Table 3:** Change in IMT_mean_ (mm) (95% Cl) over 7-years by tertiles of serum carotenoid concentrations, the Kuopio Ischaemic Heart Disease Risk Factor (KIHD) study.

	Tertiles of carotenoids			
Carotenoid	1	2	3	p-value^a^
Lycopene (µmol/L)	≤0.09	0.10–0.20	>0.20	
Model 1^b^ (change in IMT_mean_ (mm))	0.112 (0.093–0.13)	0.092 (0.073–0.11)	0.085 (0.067–0.103)	0.048
Model 2^c^ (change in IMT_mean_ (mm))	0.116 (0.098–0.135)	0.093 (0.075–0.111)	0.086 (0.068–0.104)	0.032
Model 3^d^ (change in IMT_mean_ (mm))	0.116 (0.098–0.135)	0.094 (0.075–0.112)	0.087 (0.068–0.105)	0.029
α-Carotene (µmol/L)	≤0.07	0.08–0.11	>0.11	
Model 1^b^ (change in IMT_mean_ (mm))	0.113 (0.095–0.13)	0.094 (0.075–0.114)	0.081 (0.063–0.099)	0.012
Model 2^c^ (change in IMT_mean_ (mm))	0.117 (0.10–0.135)	0.094 (0.074–0.113)	0.083 (0.065–0.10)	0.008
Model 3^d^ (change in IMT_mean_ (mm))	0.117 (0.10–0.135)	0.094 (0.075–0.114)	0.083 (0.065–0.101)	0.007
β-Carotene (µmol/L)	≤0.26	0.27–0.41	>0.41	
Model 1^b^ (change in IMT_mean_ (mm))	0.106 (0.087–0.124)	0.097 (0.080–0.115)	0.086 (0.068–0.104)	0.135
Model 2^c^ (change in IMT_mean_ (mm))	0.105 (0.086–0.124)	0.101 (0.083–0.119)	0.088 (0.070–0.107)	0.233
Model 3^d^ (change in IMT_mean_ (mm))	0.105 (0.086–0.124)	0.101 (0.083–0.119)	0.089 (0.070–0.108)	0.254

a Adjusted p-value from covariance analysis.

b Adjusted for age, examination year, ultrasound sonographer and mean of IMT_mean_.

c Adjusted for Model 1+BMI, SBP, smoking, physical activity, serum LDL cholesterol, family CHD history and drug for hypertension.

d Adjusted for Model 2+hs-CRP (Model 3).

Abbreviations as in Table 1.

**Table 4:** Change in IMT_max_ (mm) (95% Cl) over 7-years by tertiles of serum carotenoid concentrations, the Kuopio Ischaemic Heart Disease Risk Factor (KIHD) study.

	Tertiles of carotenoids			
Carotenoid	1	2	3	p-value^a^
Lycopene (µmol/L)	≤0.09	0.10–0.20	>0.20	
Model 1^b^ (change in IMT_max_ (mm))	0.109 (0.081–0.138)	0.072 (0.044–0.101)	0.052 (0.025–0.080)	0.006
Model 2^c^ (change in IMT_max_ (mm))	0.113 (0.084–0.141)	0.074 (0.045–0.102)	0.054 (0.026–0.083)	0.006
Model 3^d^ (change in IMT_max_ (mm))	0.113 (0.085–0.142)	0.075 (0.046–0.103)	0.055 (0.026–0.083)	0.005
α-Carotene (µmol/L)	≤0.07	0.08–0.11	>0.11	
Model 1^b^ (change in IMT_max_ (mm))	0.110 (0.083–0.137)	0.077 (0.048–0.107)	0.045 (0.018–0.072)	0.001
Model 2^c^ (change in IMT_max_ (mm))	0.112 (0.085–0.139)	0.077 (0.047–0.106)	0.050 (0.022–0.077)	0.002
Model 3^d^ (change in IMT_max_ (mm))	0.113 (0.084–0.140)	0.078 (0.048–0.108)	0.050 (0.022–0.077)	0.002
β-Carotene (µmol/L)	≤0.26	0.27–0.41	>0.41	
Model 1^b^ (change in IMT_max_ (mm))	0.107 (0.078–0.135)	0.079 (0.051–0.106)	0.048 (0.020–0.076)	0.004
Model 2^c^ (change in IMT_max_ (mm))	0.105 (0.076–0.134)	0.081 (0.054–0.108)	0.054 (0.025–0.083)	0.018
Model 3^d^ (change in IMT_max_ (mm))	0.105 (0.076–0.135)	0.081 (0.054–0.109)	0.055 (0.025–0.084)	0.019

a Adjusted p-value from covariance analysis.

b Adjusted for age, examination year, ultrasound sonographer and mean of IMT_max_.

c Adjusted for Model 1 + BMI, SBP, smoking, physical activity, serum LDL-c, family CHD history and drug for hypertension.

d Adjusted for Model 2+hs-CRP.

Abbreviations as in Table 1.

Regression analysis showed that after adjustment for age, examination year, ultrasound sonographer, smoking and IMT_max_, on average, for every 0.01 µmol/L increase of serum lycopene, α-carotene and β-carotene, IMT_max_ progression was decreased by 1.84, 1.86 and 1.85 µm/7-years, respectively.

## Discussion

The primary finding of our population-based study is that high serum concentrations of lycopene, α-carotene and β-carotene are associated with reduced IMT_max_ progression during 7-year follow-up. Furthermore, the high serum concentrations of lycopene and α-carotene were related to reduced IMT_mean_ progression.

There are only a few previous studies that have examined the role of carotenoids in the progression of carotid intima-media thickness [9], [10]. Epidemiological in vitro and in vivo experiments suggest that dietary lutein may be a potent carotenoid against the progression of atherosclerosis in humans [9]. In another study, plasma levels of lutein, zeaxanthin, β-cryptoxanthin and α-carotene were associated with a reduced progression of carotid IMT [10]. In our previous study we observed that high plasma concentrations of β-cryptoxanthin, lycopene and α-carotene are associated with decreased carotid atherosclerosis in elderly men [8]. In the Bruneck study, high plasma levels of α- and β-carotene were related to a lower risk of atherosclerosis [20]. A strong inverse relationship between lycopene and α-carotene plasma levels and the presence of carotid atherosclerosis was found in Manfredonia study [21]. Another study suggests that among elderly men with high plasma levels of antioxidants (such as β-carotene) have thinner artery walls and little or no plaques [22]. Inverse association between serum/plasma lycopene levels and CCA-IMT were observed in some previous studies [12], [23]. However, no association was found between plasma concentrations of carotenoids and preclinical atherosclerosis in middle-aged women [24]. It has been shown that patients with coronary artery disease, independent of clinical settings, had lower plasma levels of xantophyll carotenoids than healthy controls [25].

Oxidative stress characterized by oxidative modification of LDL-c and proteins is thought to play an important role in the initiation of atherosclerosis. Overproduction of reactive oxygen species (ROS) under pathophysiologic conditions leads to the oxidative stress. Several studies have shown that foods rich in carotenoids may reduce the risk of atherosclerosis by protecting LDL-c from oxidative modification [26]. Antioxidant mechanism of carotenoids is based on conjugated double bond system by scavenging two types of ROS: singlet molecular oxygen and peroxyl radicals to terminate injurious chain reactions [27]. β-Carotene, zeaxanthin, β-cryptoxanthin, and α-carotene belong to the group of highly active quenchers of singlet molecular oxygen [28]. Lycopene is a potent antioxidant and the most efficient quencher of singlet molecular oxygen [29]. Imbalance of nitric oxide leads to endothelial dysfunction that is signaled by impaired endothelium-dependent vasodilation. Endothelial dysfunction may be an early marker for atherosclerosis and can be detected before structural changes to the vessel wall are apparent on angiography or ultrasound [30]. It has been reported that vessel walls of carotid arteries are more elastic with subjects, whose diet is rich in carotenoids [31].

Elevated concentration of hs-CRP is associated with the risk of atherosclerotic events in general populations and is suggested to be as a major cardiovascular risk factor [32]. CRP is shown to interact with LDL, which migrates through the endothelium and become oxidatively modified by local ROS [32], [33]. Consistent with previous studies [10], [34], serum levels of carotenoids were inversely related to hs-CRP in our study. Progression of IMT_mean_ and IMT_max_ did not change considerably after further adjustment for hs-CRP suggesting that progression may not be affected by confounding of chronic inflammation.

Smoking is a known strong risk factor of atherosclerosis [35]. In the present study, smokers had lower concentrations of α-carotene than nonsmokers. This may partly be explained by differences in dietary habits, smoking and aging. We adjusted for age and smoking trying to eliminate the effect of these factors. It has been reported that free radicals of smoking deplete carotenoid levels in blood circulation and expose to oxidative stress [36].

It is hypothesized that a single compound has a little influence on the health benefits, but the combination of different antioxidants, as found in fruits and vegetables, may have pronounced health benefits. Secondly, it is shown that the combination of phenolic compounds and carotenoids led to synergistic effects by preventing human LDL-c oxidation more effectively than carotenoids alone [37]. A positive synergist effect of different type of antioxidants may have influenced the progression of carotid atherosclerosis in the present study.

The strength of this study includes its population-based design, large scale of biochemical, behavioural, health and socio-economical covariates as well as the repeated assessment of carotid IMT. There were no losses during follow-up. Unmeasured confounding factors may be associated with both serum concentrations of carotenoids and progression of atherosclerosis. There may be other components of foods containing these compounds that may explain the protective effects observed in epidemiologic studies. Our representative sample makes it possible to generalize the observed results in male populations, although the results should be confirmed in female populations.

In conclusion, this population-based study showed that high serum concentrations of lycopene, α-carotene and β-carotene at the baseline were associated with the reduced IMT progression during 7-year follow-up.

## References

[1] Steinberg D (2009) The LDL modification hypothesis of atherogenesis: An update. J Lipid Res 50: S376–381. 19011257 10.1194/jlr.R800087-JLR200 PMC2674707

[2] Itabe H (2009) Oxidative modification of LDL: Its pathological role in atherosclerosis. Clin Rev Allergy Immunol 37: 4–11. 18987785 10.1007/s12016-008-8095-9

[3] Hevonoja T, Pentikäinen MO, Hyvönen MT, Kovanen PT, Ala-Korpela M (2000) Structure of low density lipoprotein (LDL) particles: Basis for understanding molecular changes in modified LDL. Biochim Biophys Acta 1488: 189–210. 11082530 10.1016/s1388-1981(00)00123-2

[4] Poulsen HE, Jensen BR, Weimann A, Jensen SA, Sorensen M, et al (2000) Antioxidants, DNA damage and gene expression. Free Radic Res 33: S33–39. 11191273

[5] Stanner SA, Hughes J, Kelly CN, Buttriss J (2004) A review of the epidemiological evidence for the ‘antioxidant hypothesis’. Public Health Nutr 7: 407–422. 15153272 10.1079/phn2003543

[6] Riccioni G, Bucciarelli T, Mancini B, Di Ilio C, Capra V, et al (2007) The role of the antioxidant vitamin supplementation in the prevention of cardiovascular diseases. Expert Opin Investig Drugs 16: 25–32. 10.1517/13543784.16.1.25 17155851

[7] Steinberg D, Parthasarathy S, Carew TE, Khoo JC, Witztum JL (1989) Beyond cholesterol modifications of low-density lipoprotein that increase its atherogenicity. N Engl J Med 320: 915–924. 2648148 10.1056/NEJM198904063201407

[8] Karppi J, Kurl S, Laukkanen JA, Rissanen TH, Kauhanen J (2011) Plasma carotenoids are related to intima-media thickness of the carotid artery wall in men from Eastern Finland. J Intern Med 270: 478–485. 21575084 10.1111/j.1365-2796.2011.02401.x

[9] Dwyer JH, Navab M, Dwyer KM, Hassan K, Sun P, et al (2001) Oxygenated carotenoid lutein and progression of early atherosclerosis: The Los Angeles atherosclerosis study. Circulation 103: 2922–2927. 11413081 10.1161/01.cir.103.24.2922

[10] Dwyer JH, Paul-Labrador MJ, Fan J, Shircore AM, Merz CN, et al (2004) Progression of carotid intima-media thickness and plasma antioxidants: The Los Angeles atherosclerosis study. Arterioscler Thromb Vasc Biol 24: 313–319. 14656738 10.1161/01.ATV.0000109955.80818.8a

[11] Zou Z, Xu X, Huang Y, Xiao X, Ma L, et al (2011) High serum level of lutein may be protective against early atherosclerosis: The Beijing atherosclerosis study. Atherosclerosis 219: 789–793. 21872250 10.1016/j.atherosclerosis.2011.08.006

[12] Rissanen TH, Voutilainen S, Nyyssönen K, Salonen R, Kaplan GA, et al (2003) Serum lycopene concentrations and carotid atherosclerosis: The Kuopio Ischaemic Heart Disease Risk Factor Study. Am J Clin Nutr 77: 133–138. 12499332 10.1093/ajcn/77.1.133

[13] Riccioni G, Scotti L, Di Ilio E, Bucciarelli V, Ballone E, et al (2011) Lycopene and preclinical carotid atherosclerosis. J Biol Regul Homeost Agents 25: 435–441. 22023768

[14] Salonen JT (1988) Is there a continuing need for longitudinal epidemiologic research? The kuopio ischaemic heart disease risk factor study. Ann Clin Res 20: 46–50. 3408213

[15] Salonen JT, Salonen R (1993) Ultrasound B-mode imaging in observational studies of atherosclerotic progression. Circulation 87: II56–65. 8443925

[16] Salonen JT, Korpela H, Salonen R, Nyyssönen K (1993) Precision and reproducibility of ultrasonographic measurement of progression of common carotid artery atherosclerosis. Lancet 341: 1158–1159. 10.1016/0140-6736(93)93184-3 8097848

[17] Selzer RH, Hodis HN, Kwong-Fu H, Mack WJ, Lee PL, et al (1994) Evaluation of computerized edge tracking for quantifying intima-media thickness of the common carotid artery from B-mode ultrasound images. Atherosclerosis 111: 1–11. 7840805 10.1016/0021-9150(94)90186-4

[18] Thurnham DI, Smith E, Flora PS (1988) Concurrent liquid-chromatographic assay of retinol, alpha-tocopherol, beta-carotene, alpha-carotene, lycopene, and beta-cryptoxanthin in plasma, with tocopherol acetate as internal standard. Clin Chem 34: 377–381. 3342512

[19] Laukkanen JA, Kurl S, Rauramaa R, Lakka TA, Venäläinen JM, et al (2006) Systolic blood pressure response to exercise testing is related to the risk of acute myocardial infarction in middle-aged men. Eur J Cardiovasc Prev Rehabil 13: 421–428. 16926673 10.1097/01.hjr.0000198915.83234.59

[20] D'Odorico A, Martines D, Kiechl S, Egger G, Oberhollenzer F, et al (2000) High plasma levels of alpha- and beta-carotene are associated with a lower risk of atherosclerosis: Results from the Bruneck study. Atherosclerosis 153: 231–239. 11058719 10.1016/s0021-9150(00)00403-2

[21] Riccioni G, D'Orazio N, Palumbo N, Bucciarelli V, Ilio E, et al (2009) Relationship between plasma antioxidant concentrations and carotid intima-media thickness: The asymptomatic carotid atherosclerotic disease in manfredonia study. Eur J Cardiovasc Prev Rehabil 16: 351–357. 19384236 10.1097/HJR.0b013e328325d807

[22] Gale CR, Ashurst HE, Powers HJ, Martyn CN (2001) Antioxidant vitamin status and carotid atherosclerosis in the elderly. Am J Clin Nutr 74: 402–408. 11522566 10.1093/ajcn/74.3.402

[23] Gianetti J, Pedrinelli R, Petrucci R, Lazzerini G, De Caterina M, et al (2002) Inverse association between carotid intima-media thickness and the antioxidant lycopene in atherosclerosis. Am Heart J 143: 467–474. 11868053 10.1067/mhj.2002.120776

[24] Iannuzzi A, Celentano E, Panico S, Galasso R, Covetti G, et al (2002) Dietary and circulating antioxidant vitamins in relation to carotid plaques in middle-aged women. Am J Clin Nutr 76: 582–587. 12198003 10.1093/ajcn/76.3.582

[25] Lidebjer C, Leanderson P, Ernerudh J, Jonasson L (2007) Low plasma levels of oxygenated carotenoids in patients with coronary artery disease. Nutr Metab Cardiovasc Dis 17: 448–456. 17134954 10.1016/j.numecd.2006.02.006

[26] Kritchevsky SB, Tell GS, Shimakawa T, Dennis B, Li R, et al (1998) Provitamin A carotenoid intake and carotid artery plaques: The atherosclerosis risk in communities study. Am J Clin Nutr 68: 726–633. 9734754 10.1093/ajcn/68.3.726

[27] Young AJ, Lowe GM (2001) Antioxidant and prooxidant properties of carotenoids. Arch Biochem Biophys 385: 20–27. 11361018 10.1006/abbi.2000.2149

[28] Cantrell A, McGarvey DJ, Truscott TG, Rancan F, Bohm F (2003) Singlet oxygen quenching by dietary carotenoids in a model membrane environment. Arch Biochem Biophys 412: 47–54. 12646267 10.1016/s0003-9861(03)00014-6

[29] Di Mascio P, Kaiser S, Sies H (1989) Lycopene as the most efficient biological carotenoid singlet oxygen quencher. Arch Biochem Biophys 274: 532–538. 2802626 10.1016/0003-9861(89)90467-0

[30] Davignon J, Ganz P (2004) Role of endothelial dysfunction in atherosclerosis. Circulation 109: III27–32. 15198963 10.1161/01.CIR.0000131515.03336.f8

[31] Kritchevsky SB, Shimakawa T, Tell GS, Dennis B, Carpenter M, et al (1995) Dietary antioxidants and carotid artery wall thickness. The ARIC study. Atherosclerosis risk in communities study. Circulation 92: 2142–2150. 7554194 10.1161/01.cir.92.8.2142

[32] Calabro P, Golia E, Yeh ET (2009) CRP and the risk of atherosclerotic events. Semin Immunopathol 31: 79–94. 19415283 10.1007/s00281-009-0149-4

[33] Zwaka TP, Hombach V, Torzewski J (2001) C-reactive protein-mediated low density lipoprotein uptake by macrophages: Implications for atherosclerosis. Circulation 103: 1194–1197. 11238260 10.1161/01.cir.103.9.1194

[34] Kritchevsky SB, Bush AJ, Pahor M, Gross MD (2000) Serum carotenoids and markers of inflammation in nonsmokers. Am J Epidemiol 152: 1065–1071. 11117616 10.1093/aje/152.11.1065

[35] Grines CL, Topol EJ, O'Neill WW, George BS, Kereiakes D, et al (1995) Effect of cigarette smoking on outcome after thrombolytic therapy for myocardial infarction. Circulation 91: 298–303. 7805231 10.1161/01.cir.91.2.298

[36] Wei W, Kim Y, Boudreau N (2001) Association of smoking with serum and dietary levels of antioxidants in adults: NHANES III, 1988–1994. Am J Public Health 91: 258–264. 11211635 10.2105/ajph.91.2.258 PMC1446535

[37] Milde J, Elstner EF, Grassmann J (2007) Synergistic effects of phenolics and carotenoids on human low-density lipoprotein oxidation. Mol Nutr Food Res 51: 956–961. 17639513 10.1002/mnfr.200600271

